# Addition of Phospholipids Improved the Physical Stability and Fat Globule Structure of Processed Milk

**DOI:** 10.3390/foods14030375

**Published:** 2025-01-24

**Authors:** Yue Pan, Lei Zhang, Xuanfei Fu, Xiaodong Li, Lu Liu, Xuezhen Wang, Jinfeng Zhang, Wenli Zhou

**Affiliations:** 1 Food College, Northeast Agricultural University, Harbin 150030, China; yepu864084@163.com (Y.P.); zhangleihbnd@163.com (L.Z.); 15776855025@163.com (X.F.); 13363578040@163.com (X.W.); zhangfeng_1105@126.com (J.Z.); zhou17852260780@163.com (W.Z.); 2 Key Laboratory of Dairy Science, Ministry of Education, Harbin 150030, China

**Keywords:** egg yolk phospholipids, soybean phospholipids, physical stability, milk fat globule structure, infant formula

## Abstract

The manufacturing processes for infant formula disrupt the structure of phospholipid-coated milk fat globules, thereby impacting the physical stability of the system. In this study, either soybean phospholipids (SPs) or egg yolk phospholipids (EYPs) were incorporated into the milk system to reduce this damage, and their effects on the stability and fat globule structure of processed milk were examined. The findings revealed that the addition of phospholipids improved the physical stability and fat globule structure of different processed milk. In pasteurized milk, the stability constant of samples with phospholipids decreased from 0.42 to 0.37 compared to phospholipid-free milk, but no significant difference was found between the EYP and SP groups. In homogenized milk, adding EYPs resulted in a smaller particle size (870.35 nm versus 953.39 nm) and stability constant (0.28 versus 0.30) than the addition of SP. Moreover, homogenized milk added with EYPs exhibited a denser phospholipid interface film which led to a more intact fat globule structure. Consequently, the milk powder particles in the spray-dried milk supplemented with EYPs showed a more uniform distribution and smoother surface. These findings suggested that EYPs were superior to SPs in restructuring fat globules and enhancing newly formed fat globule stability during milk powder processing. This offers valuable insights for improving the physical and structural properties of dairy products, such as infant formula.

## 1. Introduction

Human milk is a natural oil-in-water emulsion, in which fat exists in the form of fat globules enveloped by a membrane known as the milk fat globule membrane (MFGM) [1]. Phospholipids constitute the fundamental framework of MFGM and represent its most important component except membrane-specific proteins [2]. The primary function of phospholipids is to maintain the stability of human milk fat globules. Phospholipids located on the surface of fat globules play a vital role in reducing the flocculation and aggregation of the fat globules [3]. Additionally, phospholipids in the MFGM also determine the structure of human milk fat globules. The human milk fat globules, which are enveloped by phospholipids skeleton, ensure that the lipids within them can be efficiently digested and absorbed [4]. Negatively charged phospholipids and lipid domains formed by different types of phospholipids facilitate binding with lipase and bile salts, thereby ensuring effective lipid digestion in infants, particularly those with immature pancreases [5].

However, in some cases, mothers cannot provide human milk, and infant formula becomes the sole food for infants. Bovine milk fat and vegetable oils are the main sources of fat in infant formulae [6]. The state of fat in the infant formula is significantly different from that of human milk. Although the fat of infant formula is also present in the form of fat globules, the fat globules are not encapsulated by MFGM, but rather by a large number of casein micelles and whey proteins [7]. The fat globules in bovine milk used for producing infant formula are enveloped by MFGM, which is similar to that of human milk, but the production process of infant formula destroys the original interface of bovine milk fat globules. The disruption of the interfacial membrane of bovine milk’s original fat globules not only leads to the loss of MFGM and its phospholipid components in infant formula, but also results in the disintegration of fat globule structure and alteration in system stability [8,9,10]. Consequently, this will impact the quality of infant formula as well as lipid nutritional properties of infant formula such as fat digestion [11]. Heat treatment, homogenization, and spray drying are the three most common processes in the production of infant formula. It has been found that when the temperature exceeds 60 °C during heat treatment, the physical state of phospholipids in the MFGM is changed and subsequently released into the serum phase [12,13]. This results in the destruction of the lipid domain and the phospholipid skeleton structure, ultimately causing the aggregation of fat globules [13]. Homogenization is another important processing procedure that affects the structure and stability of milk fat globules. Although the particle size of fat globules in milk after homogenization was significantly reduced and its stability was improved, homogenization destroyed the original structure of fat globules in milk. Homogenization changes the structure of fat globules enveloped by MFGM phospholipids into the structure of fat globules enveloped by milk proteins (casein and whey proteins), which hinders the digestion of fat [14]. Although the smaller particle size of fat globules can enhance lipid digestion, the negative effects of structural damage to the fat globules offset the benefits of particle size reduction [14]. A study by Yao et al. showed that spray drying also affects the physical, chemical, and microstructural characteristics of fat globules [15]. In brief, the manufacturing of infant formula disrupts the original properties of fat globules, resulting in significant nutritional and functional differences from natural milk. Consequently, reducing or compensating for the damage to processed milk fat globules is crucial.

Exogenous phospholipids, known for their surface activity and as an important component of biofilms, are widely used in emulsion systems to enhance the stability of the system and the structure of lipid droplets [16]. Soybean phospholipid is the most commonly used surfactant. In previous studies, soybean phospholipid was used as a membrane material to prepare homogenized milk rich in phospholipids to promote the stability of newly generated fat globules and participate in the recombination of the milk fat globule interface [17]. Therefore, the addition of exogenous phospholipid membrane materials can ameliorate the damage to the stability and structure of fat globules caused by homogenization. However, whether it can improve the damage to fat globules caused by other processing treatments, such as heat treatment and spray drying, has not been studied.

In addition to soybean phospholipids, egg yolk phospholipids are often used in emulsion systems. The study of Yu et al. showed that adding egg yolk phospholipids to infant formula improved its fat globule structure, protected the loss of MFGM protein and phospholipids, and enhanced the stability of the infant formula emulsion [18]. Exogenous phospholipids from various origins exhibit significant differences in their chemical compositions, particularly in terms of fatty acid composition, which could potentially affect their emulsifying properties, the ability to stabilize fat globules, and physiological activities [19]. A previous study has confirmed that the increase in fatty acid saturation in phospholipids contributes to greater liposome stability [20]. However, it is unclear whether this rule applies to phospholipid-stabilized milk fat globules. Therefore, it is necessary to study the effects of phospholipids from different sources on the stability of processed milk and the structure of fat globules, so as to provide a reference for the selection of phospholipids.

In this study, we investigated the effects of adding soybean phospholipids or egg yolk phospholipids on the stability and fat globule structure of processed milk treated by a series of processing processes including pasteurization, homogenization, and spray drying. The results of this study may be helpful in improving the stability and fat globule structure of processed dairy products, such as infant formula, reducing the structural damage of fat globules and the loss of functional phospholipids, and thus enhancing the quality and nutritional value of dairy products.

## 2. Materials and Methods

### 2.1. Materials and Regents

The milk was purchased from a local dairy factory (Harbin, China). Soybean phospholipids were purchased from Tiankuo Biotechnology Co., Ltd., (Shanghai, China). Egg yolk phospholipids were purchased from Mixianer Biotechnology Co., Ltd., (Xi’an, China). 1,2-dioleoyl-sn-glycero-3-phosphoethanolamine-N-(lissamine rhodamine B sulfonyl) (Rd-DOPE) fluorescent dyes were purchased from Avanti Polar Lipids Co., Ltd. (Richmond, VA, USA). Other regents were purchased from Sinopharm Group (Beijing, China).

### 2.2. The Purification of Phospholipid Materials

The purification of soybean phospholipids (SPs) and egg yolk phospholipids (EYPs) is based on the phospholipid insolubility in acetone [21]. The phospholipid material (2 g) was dissolved in acetone (10 mL) and stirred with magnetic force for 30 min. The acetone solvent in the mixture was removed by filtration, and the residual filter residue was dried in nitrogen flow. The dried sample was purified phospholipid for subsequent fatty acid detection.

### 2.3. Fatty Acid Composition Detection of SPs and EYPs

Prior to detecting the fatty acid composition of SPs and EYPs, it is necessary to convert the fatty acids in the phospholipids into their methyl esters. Acid-catalyzed transesterification was performed according to the procedure detailed by the method of Pan et al. [22]. Methylated fatty acids were detected by GC–MS. The detection conditions of SPs and EYPs were set in accordance with the method outlined by Wang et al. [23].

### 2.4. Preparation of Different Processed Milk Samples

The raw milk purchased from a local dairy factory was named RM. The pasteurized milk was prepared according to the method of Ali et al. [24]. To obtain the pasteurized milk, the raw milk was heated for 30 min at 63 °C and named PM. To obtain the SP- and EYP-fortified pasteurized milk, SPs or EYPs were added to raw milk at a concentration of 5 mg/mL. The milk samples were dispersed at 6000 rpm for 3 min with a high-speed shear disperser (IKA, Staufen, Germany). Then, the milk samples were heated for 30 min at 63 °C and named SP-PM and EYP-PM. In order to investigate the effects of adding SPs or EYPs on the stability and fat globule structure of pasteurized milk, raw milk, and PM were selected as the control groups, and SP-PM and EYP-PM were selected as the experimental groups.

The homogenized milk samples were prepared according to the method of Liu et al. [25] and Pan et al. [26]. PM, SP-PM, and EYP-PM were preheated to 65 °C, then, homogenized in a high-pressure homogenizer (AMH-3, ATS Technology, Burlington, ON, Canada) with the pressure of 20 MPa to obtain homogenized milk (HM), SP-fortified homogenized milk (SP-HM), and EYP-fortified homogenized milk (EYP-HM), respectively. In order to investigate the effects of adding SPs or EYPs on the stability and fat globule structure of homogenized milk, raw milk, and HM were selected as the control groups, and SP-HM and EYP-HM were selected as the experimental groups.

HM, SP-HM, and EYP-HM were dried using a YC-501 Laboratory spray drier (Pilotech Instrument & Equipment Co., Ltd., Shanghai, China) to obtain spray-dried milk (SDM), SP-fortified spray-dried milk (SP-SDM), and EYP-fortified spray-dried milk (EYP-SDM). The inlet air temperature was set as 160 °C, and the outlet air temperature was set as 85 °C. In order to investigate the effects of adding SPs or EYPs on the stability and fat globule structure of spray-dried milk, raw milk, and SDM were selected as the control groups, and SP-SDM and EYP-SDM were selected as the experimental groups.

### 2.5. Determination of Particle Size and Zeta–Potential of Different Processed Milk

The fat globule size distribution and zeta–potential of different processed milk samples were determined by the Nano ZS90 particle size and potential analyzer (Malvern Instruments, Malvern, UK) as described by the method of Wang et al. [27]. In order to determine the particle size, the different processed milk sample was mixed with EDTA/NaOH (35 mM, pH 7.0) to dissociate the casein micelles and then diluted 100 times with ultrapure water. The refractive of fat and water was set to 1.46 and 1.33. In order to determine the zeta–potential, the different processed milk sample was diluted 100 times by the buffer solution (20 mM imidazole, 50 mM NaCl, and 5 mM CaCl_2_). The zeta–potential determination was conducted at room temperature.

### 2.6. Determination of Stability Constant K_E_ of Different Processed Milk Samples

Stability constant K_E_ of different processed milk samples was determined according to the method described by Li et al. [3] and with slight modifications. Different processed milk sample (1 mL) was centrifuged at 4000 rpm for 15 min. The lower layer was collected and diluted 500 times with deionized water, and then its absorbance A was measured at 540 nm. The original sample that had not been centrifuged was treated according to the same operations and its absorbance A_0_ was measured at 540 nm. Calculate its stability constant K_E_ with the following formula:

$$K_{E}=|\frac{A_{0}-A}{A_{0}}|$$

### 2.7. Observation of the Fat Globule Structure of Different Processed Milk Samples

The fat globule structure of different processed milk samples was observed using a DeltaVision OMX SR ultra-high resolution microscope (Applied Precision Co., Ltd., South El Monte, CA, USA) according to the method described by Wei et al. [28]. The samples were mixed with N-(lissamine rhodamine B sulfonyl) dioleoylphosphatidylethanolamine (Rd-DOPE) at a volume ratio of 8:1. The mixtures were placed in a dark environment for 1 h. Subsequently, the mixture was dropped onto glass slides, covered, and sealed with nail polish to prevent air drying. The excitation wavelength for Rd-DOPE was set to 568 nm.

### 2.8. Observation of Surface Morphology of Spray-Dried Milk Powder

The structure of spray-dried milk powder was examined using a S-3400N scanning electron microscopy (SEM, Hitachi Co., Tokyo, Japan), adhering to the protocol described by Zhang et al. [29]. The powder was evenly distributed on double-sided adhesive tape mounted on the sample table and coated with gold in order to obtain high-quality SEM images. SEM images were captured at an acceleration voltage of 5.0 kV with magnifications of ×1000 and ×2000.

### 2.9. Statistical Analysis

We made three replications for each treatment. Each detection was performed in triplicate. One-way analysis of variance followed by the Duncan test was performed to verify differences between properties of processed milk with different phospholipids added using IBM SPSS Statistics 26. The *t*-test was applied when comparing only two datasets (i.e., the fatty acid composition of soybean phospholipids and the fatty acid composition of egg yolk phospholipids) using the same software. Differences between the mean values were regarded as significant if *p* < 0.05.

## 3. Results and Discussion

### 3.1. Fatty Acids Compositions of EYPs and SPs

As shown in Table 1, the fatty acid contents of EYPs and SPs were expressed as the percentage of a certain fatty acid to the total fatty acid. There was a significant difference in fatty acid compositions between EYPs and SPs. The fatty acids with high content associated with EYPs were C16:0 (34.2 ± 1.37%), C18:1 (30.47 ± 1.26%), and C18:2 (12.91 ± 0.38%). The most abundant fatty acids associated with SPs were C18:2 (39.32 ± 1.89%), C18:1 (22.78 ± 0.64%), and C16:0 (19.27 ± 1.20%). C15:0 and C17:0 were not detected in EYPs, while C11:0, C16:1, C20:4, and C22:6 were not detected in SP. C20:4 is essential for cognitive, neurological, and visual development [30]. In addition, we analyzed the saturation of fatty acids in EYPs and SPs. The results showed that the saturated fatty acids (SFAs) content of EYPs (48.32 ± 1.63%) was significantly higher (*p* < 0.05) than that of SPs (34.72 ± 1.30%), while the polyunsaturated fatty acids content of EYPs (17.41 ± 0.47%) was significantly lower (*p* < 0.05) than that of SPs (41.88 ± 0.53%).

Differences in the fatty acid composition of phospholipids are closely associated with nutritional and functional characteristics [19]. Interestingly, the saturation of phospholipid fatty acids affected the stability of phospholipid liposomes, and phospholipids linked with SFAs were more helpful in improving the stability of liposomes [31]. Therefore, we speculated that EYPs were more useful in enhancing the physical stability of processed milk.

### 3.2. The Impact of SP and EYP Addition on the Physical Stability and Fat Globule Structure of Pasteurized Milk

#### 3.2.1. The Effect of SP and EYP Addition on the Physical Stability of Pasteurized Milk

**Figure 1:**
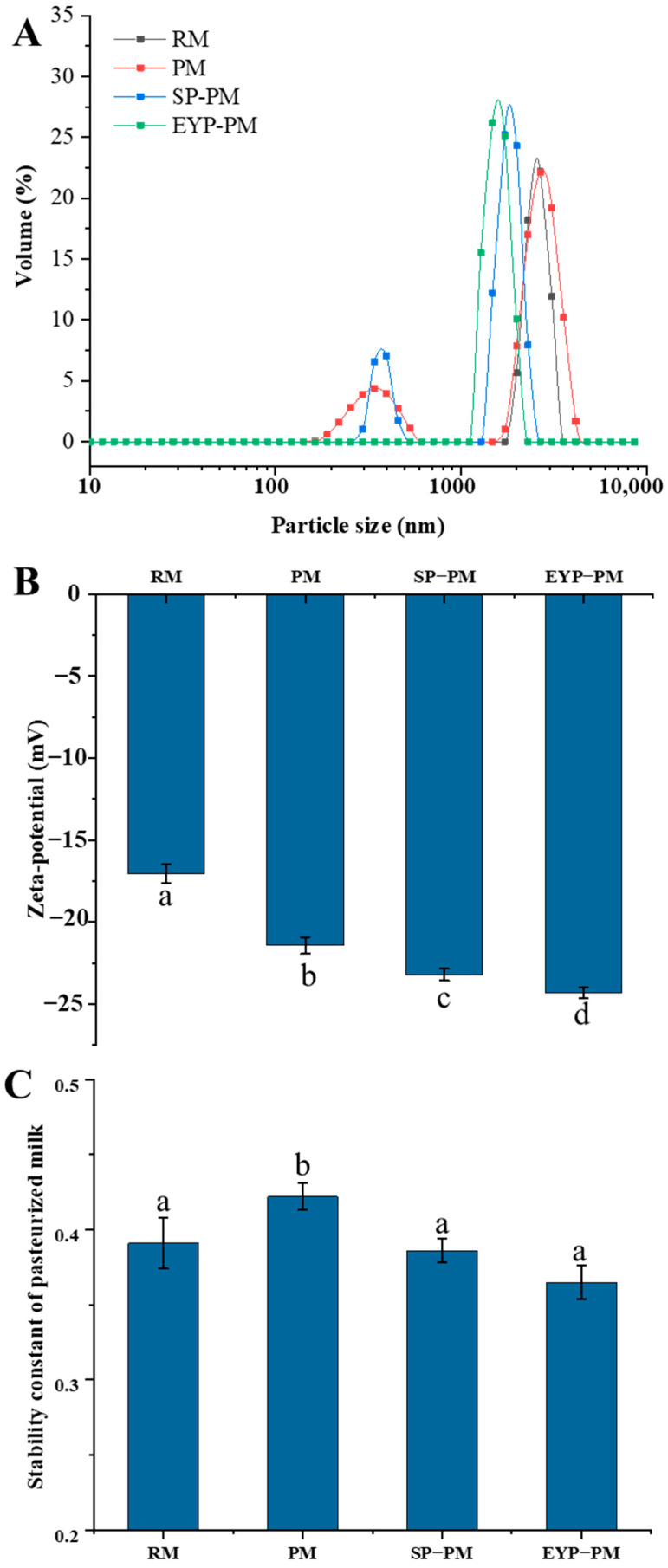
Particle size distribution (**A**), zeta–potential (**B**), and stability constant (**C**) of raw milk sample (RM), pasteurized milk sample (PM), soybean phospholipids added pasteurized milk sample (SP-PM), and egg yolk phospholipids added pasteurized milk sample (EYP-PM). a–d: different letters in the figure indicate significant differences between different treatments with 95% confidence level.

The average size of fat globules in pasteurized milk (PM) without phospholipid increased from 3.19 μm to 3.46 μm (Table S1), indicating that the heat treatment led to a slight aggregation of the fat globules. This could be attributed to the heat treatment-induced interactions between fat globules through heat-denatured proteins, which further increased the fat globules’ particle size [32]. The fat globules in the SP-PM and EYP-PM groups exhibited a significantly reduced particle size compared to those in the PM group, suggesting that the addition of exogenous phospholipids enhanced the system’s stability. The phenomenon observed in this study may be due to the exogenous phospholipids enveloping the proteins on the fat globules’ surface, which prevented thermal aggregation from occurring among fat globules. Furthermore, the particle size distribution of fat globules in the EYP-PM group is more uniform than that in the SP-PM, exhibiting a single peak distribution curve (Figure 1A). A previous study demonstrated that phospholipids enriched with saturated fatty acids enhance the hydrophobicity of liposome membranes [20]. Consequently, the higher content of saturated fatty acids in EYPs, as opposed to SPs, facilitated their adsorption onto the surface of fat globules more effectively, which in turn significantly minimized the aggregation of fat globules.

For the SP-PM and EYP-PM groups, adding phospholipid further increased the absolute value of the zeta–potential of pasteurized milk compared to the PM group (Figure 1B). In addition, the negative potential of the EYP-PM group is significantly higher than that of the SP-PM group. It can be hypothesized that these exogenous phospholipids, due to their amphiphilic and negatively charged nature, are absorbed onto the surface of milk fat globules leading to a more negative surface charge. Additionally, hydrophobic interactions enable phospholipids to bind with proteins on the fat globule surface resulting in an increased exposure of alkaline amino acid residues and leading to an increase in negative potential [33]. Previous research indicates that EYPs have a greater affinity for binding to MFGM proteins compared to SPs [34]. As a result, the zeta–potential of the FYP-PM group is significantly higher than that of the SP-PM group (*p* < 0.05), indicating stronger electrostatic repulsion and improved stability.

The stability constant of EYP-PM was not significantly different from that of RM (*p* > 0.05), which indicated that the decrease in stability caused by heat treatment could be improved by adding EYL (Figure 1C). Heat treatment leads to an increase in protein adsorption on the surface of fat globules, causing them to resemble large protein particles to some extent. Therefore, any reaction that induces protein particle aggregation, such as heat treatment, will result in a decrease in system stability. The addition of phospholipids can significantly reduce the adsorption of milk protein at the interface of fat globules. Therefore, adding exogenous phospholipids helps improve the physical stability of pasteurized milk. However, there was no statistically significant difference in the stability constant between pasteurized milk with SPs and EYPs (*p* > 0.05).

#### 3.2.2. The Effect of SP and EYP Addition on the Fat Globule Structure of Pasteurized Milk

**Figure 2:**
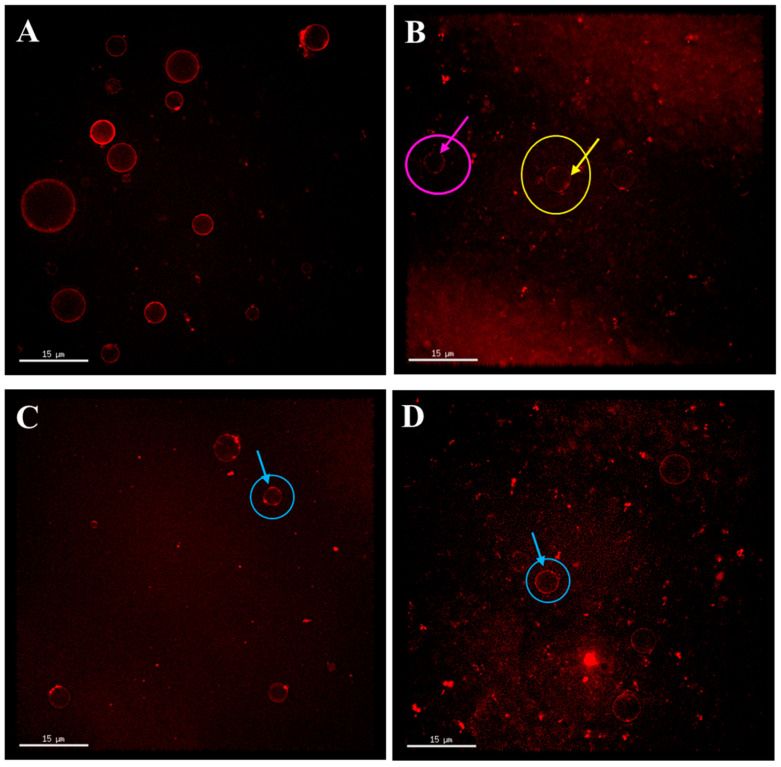
Microstructure of fat globules in raw milk sample (**A**), pasteurized milk sample (**B**), soybean phospholipids added pasteurized milk sample (**C**), and egg yolk phospholipids added pasteurized milk sample (**D**). The pink arrow and circle indicate the area that is not dyed by fluorescent dye. The yellow arrow and circle indicate a loose ring structure. The blue arrow and circle indicate a more complete ring structure.

The phospholipids and bilayers in the milk fat globule membrane are the main components and structures for stabilizing milk fat globules. The fluorescent probe Rd-DOPE can bind to the phospholipid bilayer [35]. Therefore, Rd-DOPE was used to label fat globules in this study to study the effect of adding SPs and EYPs on the fat globule structure of pasteurized milk, and the results are shown in Figure 2. In the RM group, the fat globules were uniformly stained with Rd-DOPE fluorescent dye, showing a complete red ring structure (Figure 2A), indicating that the original milk fat globules were fully covered with phospholipids. After pasteurization, the fat globules also exhibited a phospholipid-encapsulated structure, but the fluorescent ring structure of the stained phospholipids was relatively loose (indicated by the yellow arrow in Figure 2B). This may be due to the fact that when the heating temperature is higher than 60 °C, the physical state of the phospholipids in the outer layer of the fat globules changes, and the phospholipids are gradually detached from the surface of the fat globules into the serum phase, thereby modifying the arrangement of phospholipids surrounding the fat globule [15,36]. There were non-fluorescent regions observed outside the fat globules of PM (as indicated by the pink arrow in Figure 2B), which may result from the loss of phospholipids on the surface of fat globules due to pasteurization [10]. Figure 2C,D illustrate the structure of fat globules in pasteurized milk supplemented with SPs and EYPs, respectively. It can be found that the compactness of the fluorescent rings increased following the addition of phospholipids, resulting in a reduction in non-fluorescent areas surrounding the fat globules (as indicated by the blue arrow). Overall, the incorporation of exogenous phospholipids rendered the fat globule structure of pasteurized milk similar to that of raw milk.

### 3.3. The Impact of SP and EYP Addition on the Physical Stability and Fat Globule Structure of Homogenized Milk

#### 3.3.1. The Effect of SP and EYP Addition on the Physical Stability of Homogenized Milk

**Figure 3:**
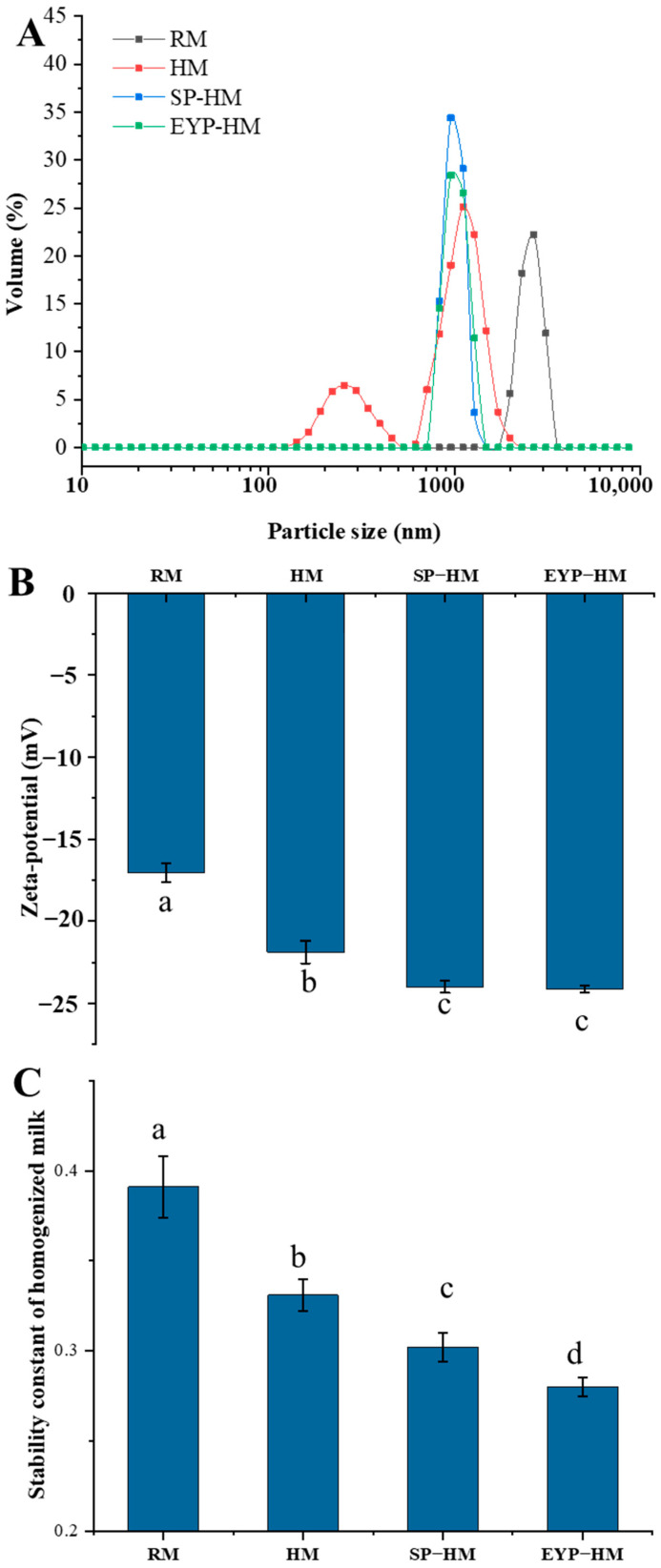
Particle size distribution (**A**), zeta–potential (**B**), and stability constant (**C**) of raw milk sample (RM), homogenized milk sample (HM), soybean phospholipids added homogenized milk sample (SP-HM), and egg yolk phospholipids added homogenized milk sample (EYP-HM). a–d: different letters in the figure indicate significant differences between different treatments with 95% confidence level.

As shown in Table S2, the volume-average particle size of fat globules decreased from 3.19 μm (RM) to 1.03 μm (HM) following homogenization. With the incorporation of SPs and EYPs, the particle size of fat globules in homogenized milk was reduced to less than 1 μm. The EYL-HM group exhibited the smallest fat globule particle size at 0.87 μm. Furthermore, the peak width of the particle size distribution curves for SL-HM and EYL-HM was narrower compared to HM, indicating that these systems were more uniform (Figure 3A). As shown in Figure 3B, the zeta–potential decreased from −17.04 mV to −21.87 mV after homogenization. Compared with the HM group, the fat globules’ surface of SL-HM and EYL-HM carried more negative charges, with values of −23.97 mV and −24.12 mV, respectively. The results of the stability constant (Figure 3C) were consistent with the results of particle size, and homogenization enhanced the stability of milk. Compared with RM, the stability constant of HM was significantly reduced by 0.06 (*p* < 0.05). The addition of SPs and EYPs further reduced the stability constant of homogenized milk, and the stability constant of the EYL-HM group was the smallest, which was 0.28. Phospholipids, recognized as natural surfactants, possess the capacity to reduce interfacial tension. Studies have demonstrated that various types of phospholipids exhibit differing abilities to lower oil–water interfacial tension [37]. Specifically, EYPs yield a lower oil–water interfacial tension compared to SPs, and emulsions stabilized by EYPs display comparatively greater stability [37]. This reason may elucidate why the physical stability of EYL-HM surpasses that of SP-HM.

#### 3.3.2. The Effect of SP and EYP Addition on the Fat Globule Structure of Homogenized Milk

**Figure 4:**
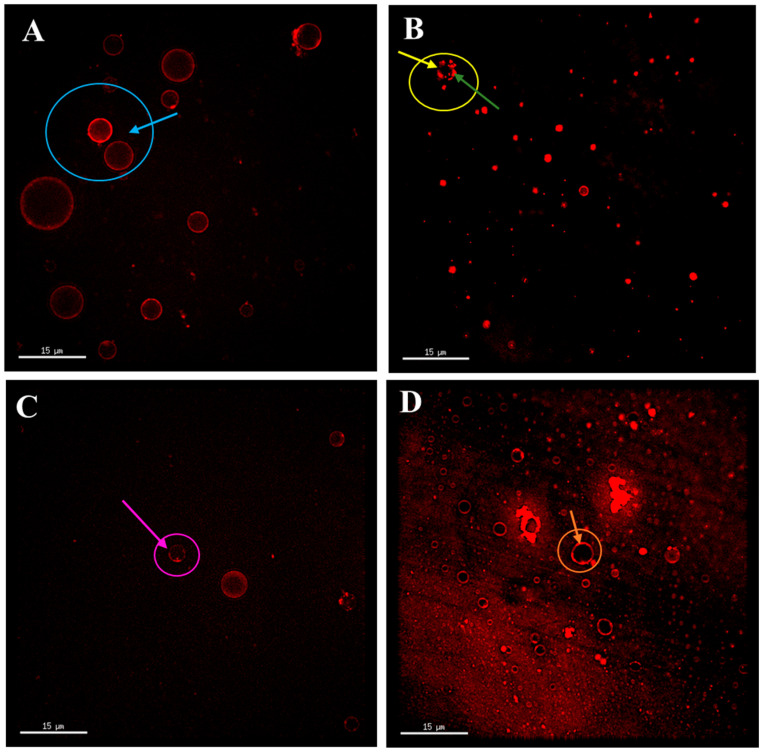
Microstructure of fat globules in raw milk sample (**A**), homogenized milk sample (**B**), soybean phospholipids added homogenized milk sample (**C**), and egg yolk phospholipids added homogenized milk sample (**D**). The blue arrow and circle indicate the complete fat globule structure. The yellow arrow indicates the area not covered by phospholipids. The green arrow indicates the area covered by phospholipids. The pink arrow and circle indicate the fat globule partially covered by phospholipids. The orange arrow and circle indicate the fat globule completely covered by phospholipids.

As shown in Figure 4, homogenization disrupted the original architecture of fat globules; however, a portion of the initial phospholipid membrane structure was preserved (as indicated by the green arrow), while some serum phase proteins that could not be stained red by fluorescent probes were found to cover the surface of the fat globules (as indicated by the yellow arrow). Studies have shown that homogenization has an adverse effect on fat digestion by damaging the fat globule structure [14]. The milk fat globule membrane phospholipids interface of raw milk fat globules is more conducive to combining lipase and bile salts than the protein interface of homogenized milk fat globules [38]. Following the addition of SPs, the structural integrity of fat globules in homogenized milk was enhanced, exhibiting both intact phospholipid-encapsulated fat globules and those partially encapsulated by phospholipids (as indicated by the pink arrow).

Upon the introduction of EYPs, nearly all fat globules displayed complete phospholipid rings (as indicated by the orange arrow), suggesting that EYPs adsorb more effectively onto the increased surface area of fat globules resulting from homogenization compared to SPs, thereby preserving the integrity of the fat globule structure. Compared to SPs, the proportion of saturated fatty acids in EYPs is higher (Table 1), which reduces the trans conformation of the phospholipid hydrocarbon chain and leads to the formation of a dense arrangement of emulsifying film on the surface of milk fat globules. This may be the main reason why the phospholipid-wrapped milk fat globules in the EYP-HM group were more intact than those in the SP-HM group. Yu et al. also demonstrated that the incorporation of EYPs reduced the levels of whey protein and casein at the fat globule interface in infant formula emulsions, while simultaneously increasing the content of interfacial phospholipids [18]. This modification enhanced the structural integrity of fat globules in infant formula emulsions, rendering their structure more akin to that of natural human milk observed by Wei et al. [39]. Therefore, the addition of EYPs is more beneficial than the addition of SPs in improving the fat globule structure of homogenized milk.

### 3.4. The Impact of SP and EYP Addition on the Physical Stability and Fat Globule Structure of Spray-Dried Milk

#### 3.4.1. The Effect of SP and EYP Addition on the Physical Stability of Reconstituted Spray-Dried Milk

**Figure 5:**
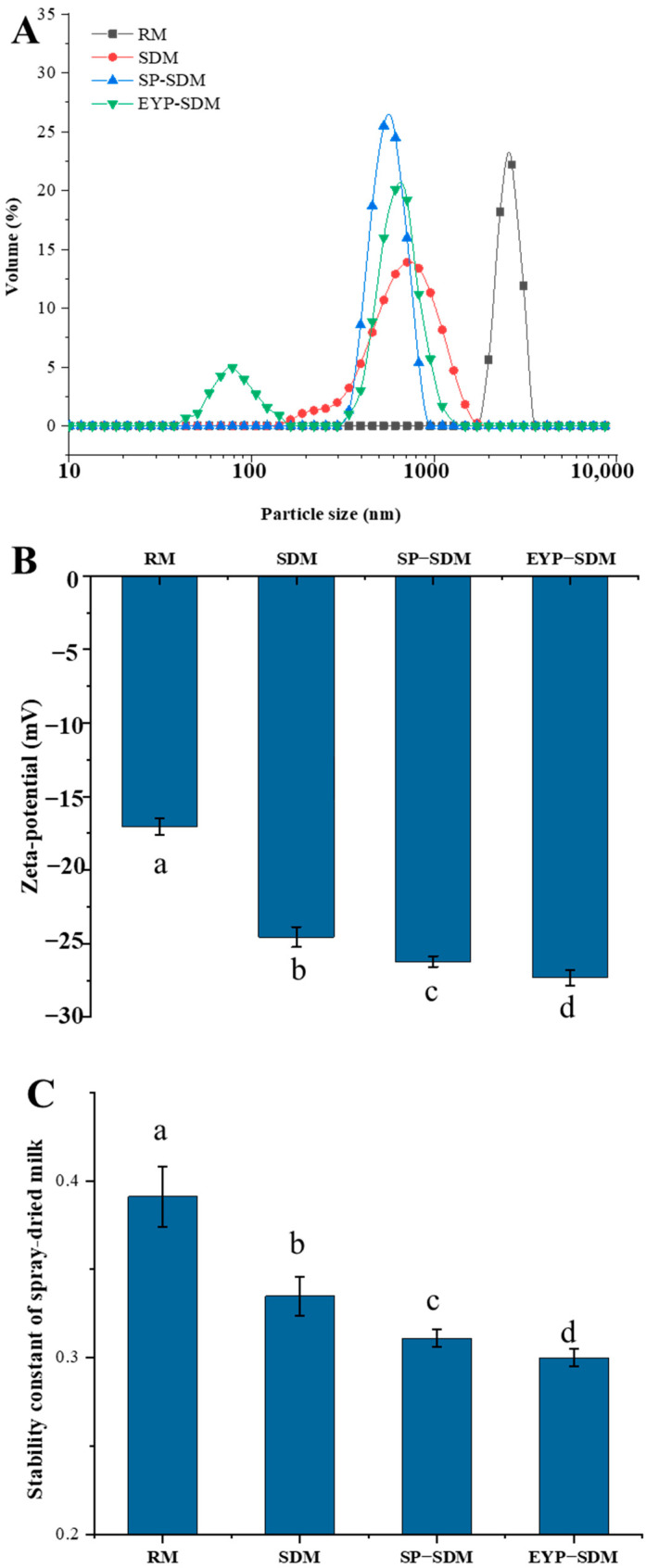
Particle size distribution (**A**), zeta–potential (**B**), and stability constant (**C**) of raw milk sample (RM), spray-dried milk sample (SDM), soybean phospholipids added spray-dried milk sample (SP-SDM), and egg yolk phospholipids added spray-dried milk sample (EYP-SDM). a–d: different letters in the figure indicate significant differences between different treatments with 95% confidence level.

As shown in Table S3, the fat globule size of reconstituted spray-dried milk was observed to decrease in the following order: SDM (635.25 nm) > SP-SDM (535.30 nm) > EYP-SDM (492.53 nm). The size distribution of SP-SDM was unimodal while that of EYP-SDM was bimodal (Figure 5A). The zeta–potentials of RM, SDM, SP-SDM, and EYP-SDM were –17.04 mV, –24.55 mV, –26.21 mV, and –27.3 mV, respectively (Figure 4B); this observation highlights that the absolute value of zeta–potential increased after the milk underwent spray drying process. This finding aligns with results from stability constants presented in Figure 5C; specifically, the stability constants for SP-SDM (0.31) and EYP-SDM (0.30) were significantly lower than that for SDM (0.34). Therefore, the addition of EYPs is more beneficial than the addition of SPs in improving the physical stability of reconstituted spray-dried milk. Previous research has demonstrated that the inclusion of phospholipids can interact with proteins within the system, effectively protecting emulsion lipid droplets against aggregation and thereby enhancing the physical stability of the system [40].

#### 3.4.2. The Effect of SP and EYP Addition on the Fat Globule Structure of Reconstituted Spray-Dried Milk

**Figure 6:**
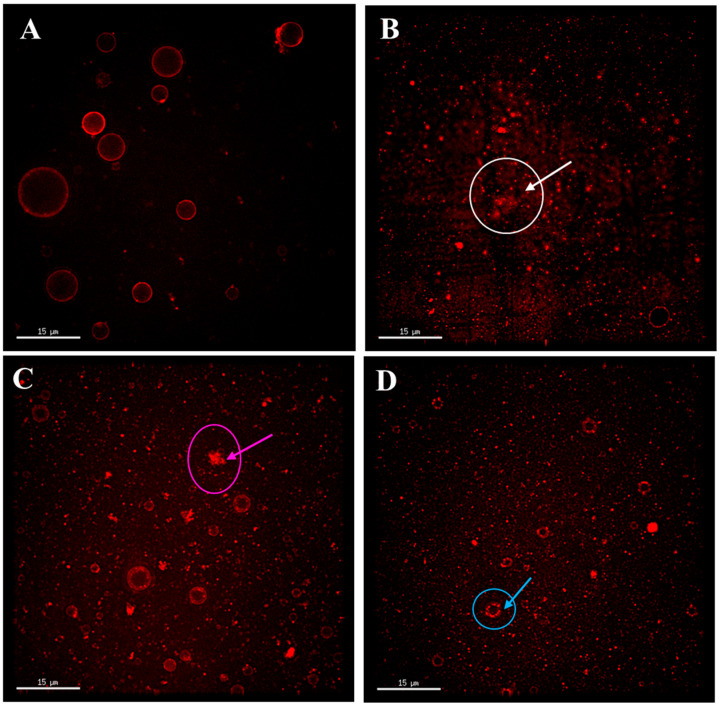
Microstructure of fat globules in raw milk sample (**A**), spray-dried milk sample (**B**), soybean phospholipids added spray-dried milk sample (**C**), and egg yolk phospholipids added spray-dried milk sample (**D**). The white arrow and circle indicate the fat globules lacking phospholipid membrane structure. The pink arrow and circle indicate the aggregated phospholipids. The blue arrow and circle indicate the ring-shaped phospholipid membrane structure.

The fat globule structure images of spray-dried milk are shown in Figure 6. Before spray drying, the fat globule in RM exhibited a ring-shaped phospholipid membrane structure. However, some fat globules in spray-dried milk lacked the phospholipid membrane structure, as pointed out by the white arrow in Figure 6B. The addition of SPs and EYPs to raw milk before homogenization resulted in an increased ring-shaped phospholipid and more obvious phospholipid membrane structure, contributing to the physical stability of the milk system. Prior studies have indicated that the addition of exogenous lecithin effectively improves the encapsulation efficiency of fat globules, thereby enhancing the structure of fat globules [41]. In contrast, the ring-shaped phospholipid membrane structure in EYP-SDM was more dense and complete than that in SP-SDM (indicated by the blue arrow in Figure 6C,D). In addition, the flocculation and coalescence of the phospholipid of SP-SDM was more severe than that of EYP-SDM (indicated by the pink arrow in Figure 6C). These observations underscore the addition of EYPs may improve the natural ring-shaped phospholipid membrane structure in spray-dried milk more effectively than EYP.

#### 3.4.3. The Effect of SP and EYP Addition on the Microstructure of Milk Powder

**Figure 7:**
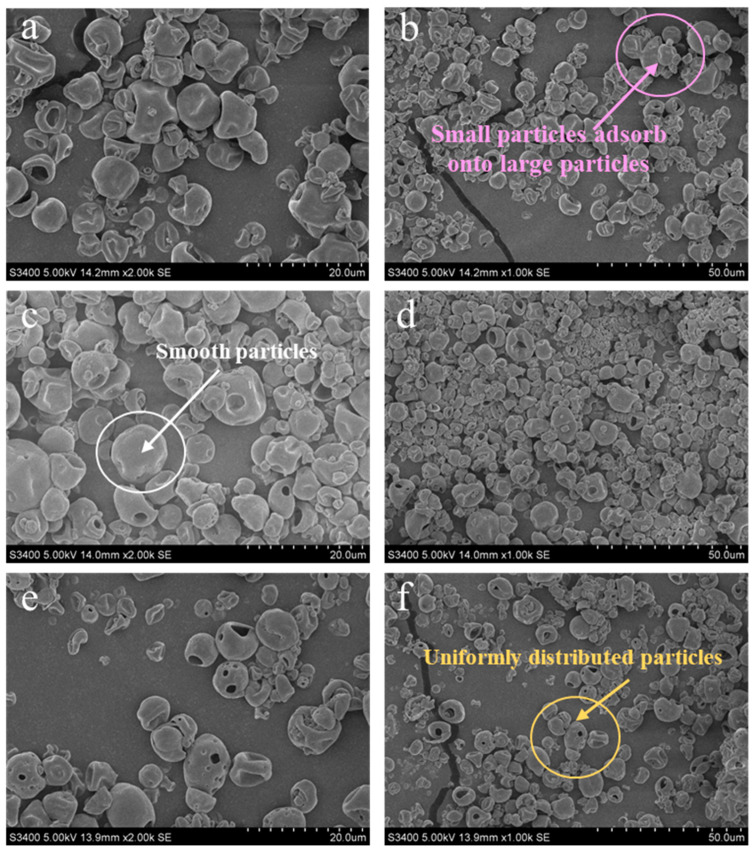
Microstructure of milk powder particles in spray-dried milk powder without phospholipid added (**a**,**b**), spray-dried milk powder added with soybean phospholipids (**c**,**d**), and spray-dried milk powder added with egg yolk phospholipids (**e**,**f**).

As shown in Figure 7a,b, the particle size of milk powder in the SDM group was uneven, with numerous smaller-sized particles adhering to the larger large-sized particles. The larger particles in the SDM group with a diameter of approximately 10 μm, and the smaller particles were about 5 μm. This may be due to the homogenization treatment of raw milk before spray drying, which destroyed the original fat globules membrane interface, made the natural fat globule structure disintegrate, and caused the electrostatic repulsion and interfacial tension between fat globules to become extremely weak, so as to aggregate and merge into larger fat globules [42]. In addition, because fine powder will be produced during spray drying, the final milk powder presents different sizes. However, with the addition of SPs and EYPs, the milk powder exhibited a smaller, rounder, and smoother particle (Figure 7c,e). This is because the phospholipid added to the milk before homogenization causes them to be adsorbed onto the fat globules’ surface along with the milk protein as a surfactant component, forming an interface membrane similar to the natural fat globule, which plays a role in stabilizing fat globules [17]. Consequently, this reduces the size of fat globules and ensures the uniform distribution of particles. The particle size of EYP-SDM was more uniform than that of SP-SDM (Figure 7d,f). A more uniform size distribution was observed in EYP-SDM, which could be attributed to the milk fat globule surface being loaded with more negative charges after the addition of EYPs. This resulted in enhanced electrostatic repulsion among fat globules, preventing their aggregation and ultimately obtaining a uniform and stable milk powder.

## 4. Conclusions

The physical stability and fat globule structure of processed milk were optimized by the addition of soybean phospholipids (SPs) and egg yolk phospholipids (EYPs). The amphiphilicity and negative charge characteristics of SPs and EYPs have made significant contributions to improving the physical stability of processed milk. The added phospholipids can be adsorbed onto the surface of milk fat globules, increasing electrostatic repulsion between them, thus preventing aggregation. It is worth noting that EYPs have a stronger ability to improve the physical stability of homogenized milk and spray-dried milk compared to SPs. This result is mainly due to the higher content of saturated fatty acids in EYPs. The increased amount of saturated fatty acids in EYPs leads to the formation of a dense arrangement of emulsifying film on the surface of milk fat globules, resulting in a more complete and compact structure of phospholipid-coated fat globule when adding EYPs compared to SPs. Furthermore, EYPs, which are rich in saturated fatty acids, also resulted in smoother and more uniform particle formation in spray-dried milk powder. These findings have potential applications for optimizing the physical stability and structure of dairy products, especially infant formula.

## Figures and Tables

**Table 1:** Fatty acids of compositions of egg yolk phospholipids and soybean phospholipids (% of total fatty acids).

Fatty Acid	Egg Yolk Phospholipids	Soybean Phospholipids	*p*-Value	Significance
C6:0	0.48 ± 0.01	3.04 ± 0.26	0.003382	**
C10:0	0.08 ± 0.01	1.51 ± 0.72	0.075079	NS
C11:0	0.28 ± 0.02	0.00 ± 0.00	0.001696	**
C12:0	2.35 ± 0.39	1.85 ± 0.03	0.155764	NS
C14:1	1.15 ± 0.18	0.62 ± 0.00	0.036364	*
C14:0	2.50 ± 0.53	2.93 ± 0.01	0.295126	NS
C16:1	2.65 ± 0.82	0.00 ± 0.00	0.030465	*
C16:0	34.2 ± 1.37	19.27 ± 1.20	0.000159	**
C17:0	0.00 ± 0.00	0.28 ± 0.02	0.001696	**
C18:2	12.91 ± 0.38	39.32 ± 1.89	0.001185	**
C18:1	30.47 ± 1.26	22.78 ± 0.64	0.002646	**
C18:0	8.71 ± 1.45	5.81 ± 0.48	0.030255	*
C20:4	0.64 ± 0.04	0.00 ± 0.00	0.001300	**
C21:0	2.56 ± 0.32	2.56 ± 0.08	1.000000	NS
C22:6	1.30 ± 0.14	0.00 ± 0.00	0.003844	**
SFAs	48.32 ± 1.63	34.72 ± 1.30	0.000453	***
MUFAs	34.27 ± 0.52	23.4 ± 1.05	0.000015	***
PUFAs	17.41 ± 0.47	41.88 ± 0.53	0.000000	***

Abbreviations: SFAs = saturated fatty acids; MUFAs = monounsaturated fatty acids; PUFAs = polyunsaturated fatty acids; NS = not significant; (*) = *p* < 0.05; (**) = *p* < 0.01; (***) = *p* < 0.001.

## Data Availability

The original contributions presented in the study are included in the article/Supplementary Materials, further inquiries can be directed to the corresponding authors.

## Supplementary Materials

The following supporting information can be downloaded at https://www.mdpi.com/article/10.3390/foods14030375/s1, Table S1: Volume average particle size of RM, PM, SP-PM, and EYP-PM; Table S2: Volume average particle size of RM, HM, SP-HM, and EYP-HM; Table S3: Volume average particle size of RM, SDM, SP-SDM, and EYP-SDM.
