# Supplementary material for: Addition of Phospholipids Improved the Physical Stability and Fat Globule Structure of Processed Milk
Source: Foods. 2025 Jan 24;14(3):375. doi: 10.3390/foods14030375 (PMC11817397; doi:10.3390/foods14030375)
Supplement: Supplementary file 1 [file foods-14-00375-s001.zip › foods-3436359-supplementary.pdf]

Addition of Phospholipids Improved the Physical Stability and Fat Globule Structure of Processed Milk

Yue Pan [1,2], Lei Zhang [1,2], Xuanfei Fu [1], Xiaodong Li [1,*], Lu Liu [2,*], Xuezhen Wang [1,2], Jinfeng Zhang [1,2] and Wenli Zhou [1,2]

[1] Food College, Northeast Agricultural University, Harbin 150030, China
[2] Key Laboratory of Dairy Science, Ministry of Education, Harbin 150030, China
* Correspondence: hrblxd@163.com (X.L.); liulu89824@163.com (L.L.)

Table S1. Volume average particle size of RM, PM, SP-PM, and EYP -PM.

Sample	Volume average particle size (μm)
RM	3.19 ± 0.13 [b]
PM	3.46 ± 0.02 [a]
SP-PM	2.69± 0.16 [c]
EYP-PM	2.12± 0.11 [d]

[a], [b], [c], [d] Different letters in the same column indicate significant differences between different treatments with 95% confidence level using Duncan test.

Abbreviations: RM = raw milk; PM = pasteurized milk; SP-PM = pasteurized milk added with soybean phospholipids; EYP-PM = pasteurized milk added with egg yolk phospholipids.

Table S2. Volume average particle size of RM, HM, SP-HM, and EYP-HM.

Sample	Volume average particle size (nm)
RM	3190.38 ± 130.21[a]
HM	1030.28 ± 49.69 [b]
SP-HM	953.39 ± 76.28 [c]
EYP-HM	870.35 ± 57.43 [d]

[a], [b], [c], [d] Different letters in the same column indicate significant differences between different treatments with 95% confidence level using Duncan test.

Abbreviations: RM = raw milk; HM = homogenized milk; SP-HM = homogenized milk added with soybean phospholipids; EYP-HM = homogenized milk added with egg yolk phospholipids.

Table S3. Volume average particle size of RM, SDM, SP-SDM, and EYP-SDM.

Sample	Volume average particle size (nm)
RM	3190.38 ± 130.21[a]
SDM	635.25 ±33.79[b]
SP-SDM	535.30 ± 13.27[c]
EYP-SDM	492.53 ± 23.58[d]

[a], [b], [c], [d] Different letters in the same column indicate significant differences between different treatments with 95% confidence level using Duncan test.

Abbreviations: RM = raw milk; SDM = spray-dried milk; SP-SDM = spray-dried milk added with soybean phospholipids; EYP-SDM = spray-dried milk added with egg yolk phospholipids.
